# Regulation of Ferroptosis Sensitivity in Hepatocellular Carcinoma Cells by Lysosomal Ion Channels TPC2 and TRPML1

**DOI:** 10.3390/antiox15050618

**Published:** 2026-05-13

**Authors:** Franz Geisslinger, Victoria Gell, Finja Witt, Dawid Jaślan, Christian Grimm, Andreas Koeberle, Karin Bartel

**Affiliations:** 1 Department of Pharmacy, Ludwig-Maximilians-Universität München, 80539 Munich, Germany; 2 Michael Popp Institute, Center for Molecular Biosciences Innsbruck (CMBI), University of Innsbruck, 6020 Innsbruck, Austria; 3 Walther Straub Institute of Pharmacology and Toxicology, Faculty of Medicine, Ludwig-Maximilians-Universität München, 80336 Munich, Germany; 4 Immunology, Infection and Pandemic Research IIP, Fraunhofer Institute for Translational Medicine and Pharmacology ITMP, 80333 Munich, Germany; 5 Department of Pharmacology, Faculty of Medicine, University of Oxford, Oxford OX1 3QT, UK; 6 Institute of Pharmaceutical Sciences and Excellence Field BioHealth, NAWI Graz, University of Graz, Beethovenstraße 8, 8010 Graz, Austria; 7 Cluster for Nucleic Acid Therapeutics Munich (CNAT-M), 80333 Munich, Germany

**Keywords:** ferroptosis, hepatocellular carcinoma, lysosomes, TPC2, TRPML1, lipid peroxidation, redox biology, calcium signaling, ACSL4, oxidative stress

## Abstract

Ferroptosis is an iron-dependent, lipid peroxidation–driven form of regulated cell death that has emerged as a therapeutic vulnerability in hepatocellular carcinoma (HCC), yet the contribution of lysosomes to this process remains incompletely understood. In this study, we investigated whether lysosomal ion channels regulate ferroptosis sensitivity in HCC cells, focusing on the two-pore channel 2 (TPC2) and the transient receptor potential mucolipin 1 (TRPML1). Using pharmacological modulation, genetic knockout models, flow cytometry-based cell death and lipid peroxidation assays, lipidomics, calcium measurements, and molecular analyses across multiple HCC cell lines, we examined how these channels influence ferroptotic signaling. We show that NAADP-dependent TPC2 activity is required for efficient ferroptosis induction, whereas TPC2 loss renders HCC cells resistant to ferroptosis triggered by system Xc^−^ inhibition or glutathione peroxidase 4 (GPX4)blockade. This resistance is associated with reduced lipid peroxidation, altered calcium signaling, and selective depletion of polyunsaturated phosphatidylethanolamine species linked to decreased Acyl-CoA Synthetase Long-Chain Family Member 4 (ACSL4) expression. In contrast, TRPML1 deficiency sensitizes cells to ferroptosis and correlates with enhanced endoplasmic reticulum stress and oxidative imbalance rather than major lipid remodeling. Collectively, these findings identify lysosomal ion channels as key modulators of ferroptosis in HCC and highlight distinct mechanisms by which TPC2 and TRPML1 regulate cellular redox balance and death susceptibility.

## 1. Introduction

Ferroptosis is a regulated form of cell death driven by the iron-dependent accumulation of lipid peroxides and reactive oxygen species (ROS), fundamentally distinct from apoptosis, necroptosis, and other classical death pathways [1,2]. It arises from an imbalance between oxidative damage and cellular antioxidant defenses, particularly glutathione (GSH) and glutathione peroxidase 4 (GPX4), which normally limit lipid peroxidation [3,4]. Because ferroptosis involves iron redox chemistry and enzymatic ROS generation, it lies at the intersection of redox biology and antioxidant mechanisms, making it highly relevant to fields spanning cancer biology, neurodegeneration, cardiovascular disease, and metabolic disorders.

In cancer, evasion of regulated cell death is a hallmark that contributes to tumor progression and therapy resistance [5,6]. Ferroptosis can bypass many resistance mechanisms and has therefore emerged as a promising anti-cancer therapeutic strategy [7,8]. The major biochemical hallmark of ferroptosis is iron-dependent, uncontrolled lipid peroxidation. Multiple antioxidant systems—including the system Xc^−^/GSH/GPX4 axis—act to suppress this lipid peroxidation, highlighting the complex redox regulation underlying this process [3,7].

Recent work has implicated lysosomes as critical subcellular sites for ferroptotic lipid oxidation and redox regulation, bringing lysosomes to the center of ferroptosis research. The lysosomal lumen contains high levels of several ions, including iron and calcium, both important regulators of ferroptosis. While iron drives lipid peroxidation via the Fenton reaction [9,10], calcium levels increase upon ferroptosis induction prior to cell death [11]. As lysosomes are iron and calcium-rich in their lumen [12,13], it is essential to shed more light on the role of the lysosome in the course of ferroptotic cell death.

In that regard, calcium channels such as TRPML1 and TPC2 are permeable for a variety of ions and might regulate cation release from the lysosomal lumen upon ferroptosis induction. Hence, these channels are the focus of our research.

TPC2 is a member of the two-pore channel family, consisting of two isoforms, TPC1 and TPC2, of which TPC2 is especially important for cancer cell hallmarks [14,15,16]. For instance, TPC2 regulates cell proliferation, energy metabolism, and migration in tumor cells. TPC2 is activated by the binding of either PI(3,5)P_2_, leading to a predominant sodium release, or NAADP, leading to calcium efflux [17,18,19,20]. Although many cancer hallmarks regulated by TPC2 were identified in the past, its role in ferroptosis is not yet clear

TRPML1 is a member of the transient receptor potential mucolipin 1 family, which is ubiquitously expressed in late endosomes and lysosomes [21,22,23]. Like TPC2, it is activated by PI(3,5)P_2_, but furthermore by ROS [24,25,26]. The cation channel is conductive for a wide variety of cations, including calcium, sodium, ferrous iron, zinc, magnesium, and potassium, and has been implicated in cancer progression [27,28]. Given its involvement in iron homeostasis and oxidative stress, we investigated TRPML1’s influence on ferroptosis as a potential anti-cancer mechanism.

In this study, we investigated the role of lysosomal signaling in ferroptosis of hepatocellular carcinoma (HCC) and specifically the potential opportunity to modulate ferroptosis response by modulation of TPC2 and TRPML1. We provide evidence for distinct roles of both cation channels in modulating ferroptosis sensitivity of HCC cells, hence depicting them as crucial regulators of cellular redox balance.

## 2. Materials and Methods

### 2.1. Cell Lines

RIL-175 wt cells were a kind gift from Prof. Dr. Simon Rothenfußer (Ludwig-Maximilians-Universität, Munich, Germany). RIL-175 TPC2 KO cells were generated by Dr. Martin Müller (Ludwig-Maximilians-Universität, Munich, Germany) and previously validated [18]. Ril-175 TRPML1 KO cells were generated by Dr. Wei-Xiong Siow and previously validated [28]. HUH-7 cells were obtained from JCRB (JCRB0403). Hep3B and HepG2 cells were obtained from ATCC (LGC, Guildford, UK). All cell lines were cultivated in DMEM (Anprotec, Bruckberg, Germany) supplemented with 10% FCS (Anprotec, Bruckberg, Germany). For HUH-7 cells, all culture flasks and well plates were precoated with Collagen G to improve adhesion.

### 2.2. Compounds and Treatment

RSL3, erastin, ferrostatin-1, and arachidonic acid were obtained from Cayman Chemicals (LGC, Guildford, UK). CA-074Me, Pepstatin A, siramesine, and Z-VAD-FMK were obtained from Selleckchem (Houston, TX, USA). CP-24879, zileuton, concanamycin A, TOFA, C75, etomoxir, and GSK2387808A were obtained from MedChemExpress (Sollentuna, Sweden). Chloroquine, glutamate, Ned-19, YM-201636, and 2-deoxy glucose were obtained from Merck (Merck KGaA, Darmstadt, Germany). sg094, TPC2-A1-N, TPC2-A1-P, EDME, and ML1-SA1 were kindly provided by Prof. Dr. Franz Bracher and Dr. Marco Keller (Ludwig-Maximilians-Universität, Munich, Germany). All compounds except for arachidonic acid, chloroquine, glutamate, and 2-DG (all dissolved in demineralized water) were dissolved in DMSO and used in concentrations not exceeding a concentration of 0.1% DMSO for cell culture applications. Delipidized medium (DLM) was obtained by supplementation of 10% lipid-free serum (Anprotec, Bruckberg, Germany) to DMEM.

### 2.3. CellTiter-Blue Cell Viability Assay

Cells were seeded at a density of 1 × 10^3^ cells/well and treated as indicated. After 2 h of cell seeding, zero values were determined by incubation with CellTiter-Blue reagent for 2 h. Then, 2 h prior to the end of the experiment, cells were incubated with CellTiter-Blue reagent for 2 h. Relative proliferation was calculated as follows:

$$relative\;proliferation=\frac{inensity\left(treated\right)-intensity\left(zero\;value\right)}{intensity\;(control)-intensity\;(zero\;value)}$$

### 2.4. Flow Cytometry

Cells were plated at a density of 2.5 × 10^4^ (RIL-175 wt, TPC2 KO, and TRPML1 KO) or 5 × 10^4^ (HUH-7, HepG2, Hep3B) cells per well and were subjected to treatment 24 h after seeding. For assessment of cell death, cells were incubated with 1 mg/mL propidium iodide in PBS immediately before analysis. The population was gated for PI-positive events, which were classified as dead cells. Specific cell death was determined using the following formula: specific cell death [%] = (% dead cells (treated) − % dead cells (ctrl))/(100% − dead cells (ctrl)) × 100. Lipid peroxidation was measured using Bodipy C11 (Sigma Aldrich, St. Louis, MO, USA); cells were loaded with 5 µM Bodipy C11 30 min prior to the start of the experiment, and the Bodipy C11-positive population was gated for analysis. Intracellular calcium levels were assessed using Cal-520AM (Sigma Aldrich, St. Louis, MO, USA) (2 µM, 2 h), and ΔF/F0 values were calculated. Lipid droplet content was quantified using Bodipy 493/503 (Sigma Aldrich, St. Louis, MO, USA) (2.5 µg/mL, 30 min). Fatty acid uptake was evaluated with Bodipy C12 558/668 (Sigma Aldrich, St. Louis, MO, USA) at variable concentrations over 4 h. Mitochondrial superoxide levels were measured using MitoSOX (Sigma Aldrich, St. Louis, MO, USA) (2 µM, 20 min), while mitochondrial mass was determined using MitoTracker Green (Sigma Aldrich, St. Louis, MO, USA) (100 nM, 30 min). All samples were collected on a BD FACS Canto II (BD) (BD Biosciences, Franklin Lakes, NJ, USA) and analyzed with FlowJo (v10.10.1).

### 2.5. Free Fatty Acid Analysis

Free fatty acids were detected according to the manufacturer’s protocol (MAK044, Sigma Aldrich, St. Louis, MO, USA), as described before [29]. Briefly, cells were treated as indicated, harvested, and homogenized in 1% Triton X-100 (Sigma Aldrich, St. Louis, MO, USA) in chloroform. The organic phase was collected after centrifugation and vacuum-dried. Lipids were re-dissolved in assay buffer and incubated with the reaction mix. Absorbance was measured with an infinite F200Pro plate reader (Tecan, Männedorf, Switzerland) and is proportional to free fatty acid content.

### 2.6. Targeted Analysis of Phosphatidylethanolamines by UPLC-MS/MS [30]

Cell pellets corresponding to 2 × 10^6^ cells were collected by centrifugation (270× *g*, 5 min, 4 °C), washed with PBS, and snap-frozen in liquid nitrogen after removal of the supernatant. For lipid extraction, cells were resuspended in 150 µL PBS, followed by the addition of 365 µL of an internal standard solution (1 µL of 0.2 mM 1,2-dimyristoyl-sn-glycero-3-phosphoethanolamine (DMPE) in 364 µL methanol) and 187.5 µL chloroform. After mixing for 30 s, a second aliquot of chloroform (187.5 µL) was added, and the sample was mixed again. Phase separation was induced by the addition of 187.5 µL 0.9% NaCl solution, followed by further mixing (30 s) and centrifugation (1500× *g*, 5 min, 4 °C). The lower organic phase was collected, transferred to a fresh tube, and evaporated to dryness in a vacuum concentrator at 30 °C for 30 min. The resulting lipid film was reconstituted in 100 µL methanol and clarified by centrifugation (20,000× *g*, 5 min, 4 °C). Supernatants were diluted in methanol and centrifuged again under the same conditions prior to analysis. Chromatographic separation of phosphatidylethanolamines (PEs) was carried out using an Acquity UPLC BEH C8 column (1.7 µm, 2.1 × 100 mm; Waters, Milford, MA, USA) coupled to an ExionLC™ AD UHPLC system (Sciex, Marlborough, MA 01752, USA). The mobile phases consisted of A (water/acetonitrile, 90:10, supplemented with 2 mM ammonium acetate) and B (acetonitrile/water, 95:5, supplemented with 2 mM ammonium acetate). The flow rate was set to 0.75 mL/min. Separation was achieved using a gradient starting at 25% A/75% B, increasing to 85% B over 5 min, followed by isocratic elution at 100% B for 2 min. The column temperature was maintained at 45 °C. Detection was performed using electrospray ionization in negative mode with multiple reaction monitoring (MRM) on a QTRAP 6500+ mass spectrometer (Sciex). For each PE species, both corresponding fatty acid fragment ions were monitored, and quantification was based on the mean signal intensity of the respective transitions. Instrument parameters were set as follows: curtain gas 40 psi, collision gas medium, ion spray voltage −4500 V, source temperature 650 °C, sheath gas 55 psi, and auxiliary gas 75 psi. Compound-dependent parameters included a declustering potential of −50 V, entrance potential of −10 V, collision energy of −38 eV, and collision cell exit potential of −12 V. Total PE levels were calculated as the sum of all detected PE species. Absolute data were normalized to the internal standard DMPE, and relative abundances of individual lipid species were expressed as percentages of the total PE signal.

### 2.7. Confocal Microscopy

Confocal microscopy was carried out using a Leica TCS SP8 confocal microscope (Wetzlar, Germany) equipped with an HC PL APO CS2 63×/1.4 oil immersion objective and controlled via Las X software (v3.10) (Leica, Wetzlar, Germany). Based on the excitation spectra of the respective dyes, excitation was performed using 405 nm, 488 nm, or 561 nm laser lines. Fluorescence emission was detected using either PMT or HyD detectors. Image processing and analysis were performed in ImageJ (v1.54r). Cells were seeded, cultured, and stained in ibiTreat μ-Slides. Neutral lipid stores were labeled with Bodipy 493/503 (Sigma Aldrich, St. Louis, MO, USA) (2.5 µg/mL, 30 min). Cells were then washed twice with PBS and fixed in 4% formaldehyde in PBS for 10 min. After fixation, cells were again washed twice with PBS and mounted using FluorSave reagent (Merck Millipore, Darmstadt, Germany) together with coverslips. Nuclei were counterstained with Hoechst 33342 post-fixation (Sigma Aldrich, St. Louis, MO, USA). For image analysis, LysoTracker-positive regions were identified in ImageJ following appropriate threshold adjustment, and positive particles were subsequently quantified using the “analyze particles” function.

### 2.8. Immunoblotting

Cells were processed as described before [17]. Cells were either treated as indicated or left untreated, then harvested by centrifugation and washed twice with ice-cold PBS. For preparation of whole-cell lysates, cells were incubated in a detergent-based lysis buffer (1% NP-40, 0.1% SDS, 0.25% deoxycholate, 150 mM NaCl, 50 mM Tris-HCl in deionized water; pH 7.5). Protease inhibitor Complete (Roche, Basel, Switzerland) was added immediately prior to use. Protein concentration was determined using a Bradford assay against a BSA standard curve by measuring absorbance at 592 nm with a plate reader.

Appropriate volumes of 5× sample buffer (3.125 M Tris-HCl (pH 6.8), 50% glycerol, 5% SDS, 2% DTT, 0.025% Pyronin Y) and 1× sample buffer were used to adjust protein concentrations. Samples were then separated by SDS-PAGE (100 V for 21 min, 200 V for 40 min). Protein loading was assessed using stain-free technology, and gels were imaged using a ChemiDoc imaging system (Bio-Rad Laboratories, Hercules, CA, USA). Proteins were subsequently transferred onto PVDF membranes via tank blotting (100 V, 1.5 h, 4 °C). Membranes were washed in TBS-T and blocked with 5% BSA in TBS-T.

Target proteins were detected using specific primary antibodies, including ACLS4 (sc-271800) (Santa Cruz, Santa Cruz, CA, USA), followed by HRP-conjugated secondary antibodies and a 2.5 mM luminol-based detection solution. Imaging was performed with a ChemiDoc Touch imaging system (Bio-Rad). Data analysis was carried out in ImageLab (Bio-Rad), and band intensities were normalized to total protein loaded per lane as determined by stain-free detection.

### 2.9. Quantitative Real-Time PCR (RT-qPCR)

Total mRNA was extracted using the RNeasy Mini Kit (Qiagen, Venlo, The Netherlands) following the manufacturer’s instructions, including an on-column DNase digestion step to remove DNA contamination. Lysis Buffer RLT was supplemented with 40 μM DTT immediately before use. To enhance cell disruption, cells resuspended in buffer RLT were frozen at −80 °C for at least 30 min prior to processing. RNA yield was measured using a Nanodrop ND-100 spectrophotometer (PEQLAB Biotechnologie GmbH, Erlangen, Bavaria, Germany). Reverse transcription to cDNA was performed with the High-Capacity cDNA Reverse Transcription Kit (Applied Biosystems, Thermo Fisher Scientific, Waltham, MA, USA) according to the manufacturer’s protocol. RT-qPCR was carried out using PowerUp SYBR Green Master Mix (Applied Biosystems) on a QuantStudio 3 Real-Time PCR System (Applied Biosystems). qPCR primers were obtained from Metabion and validated by assessment of primer efficiency as well as melt curve analysis of the resulting amplification products. Results were evaluated employing the ΔΔCT method as described previously [31]. Actin served as a housekeeping gene.

### 2.10. Statistical Analyses

Experiments were performed in at least three independent replicates unless otherwise indicated, and the number of replicates is specified in the figure legends. Data are presented as mean ± standard deviation (SD) unless stated differently. Statistical analyses and non-linear regression were carried out using GraphPad Prism 9. For comparisons between two groups, statistical significance was assessed using a two-tailed Student’s t test, with Welch’s correction applied when appropriate. For comparisons involving more than two groups within a single dataset, ordinary one-way ANOVA was used, followed by either Dunnett’s or Tukey’s post hoc test. For analyses involving two independent variables across different groups, an ordinary two-way ANOVA was performed with Sidak’s or Tukey’s post hoc test, as specified in the corresponding figure legends. Differences in concentration–response curves were evaluated using the comparison of fits function in GraphPad Prism 9. Statistical significance was defined as *p* < 0.05.

### 2.11. Use of GenAI

The generative AI ChatGPT (OpenAI GPT-5.5) has been used to polish language and grammar. Further, AI has been used to improve the concision of the text or to support literature research. The authors have reviewed and edited the output and take full responsibility for the content of this publication.

## 3. Results

### 3.1. TPC2 Inhibition or TRPML1 Activation Blocks Ferroptosis Induction

**Figure 1:**
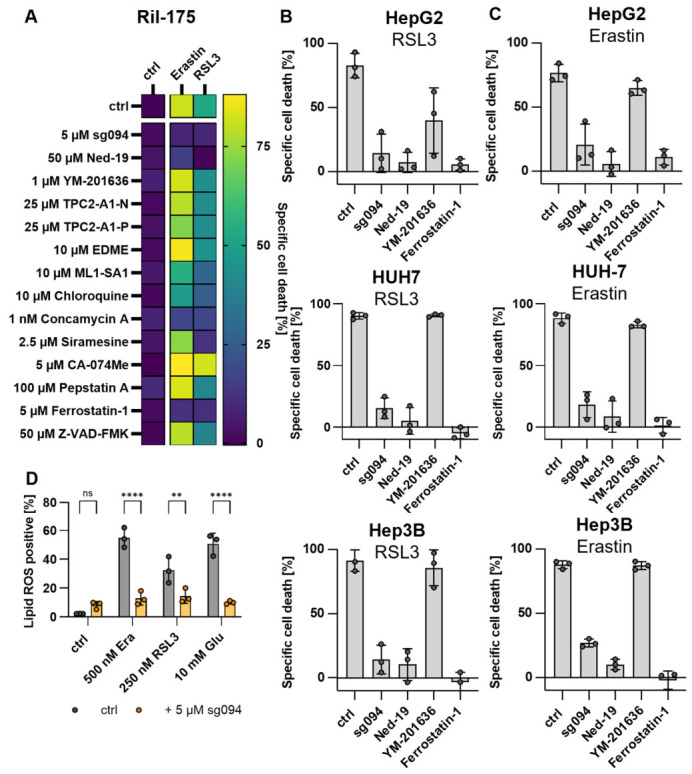
**TPC2 inhibition (or TRPML1 activation) blocks ferroptosis induction.** (**A**–**C**) Specific cell death was determined by propidium iodide staining and flow cytometry after ferroptosis induction using erastin or RSL3, respectively. (**A**) Heatmap depicting specific cell death in Ril-175 after treatment as indicated. (**B**,**C**) Bar graphs depicting cell death induction after treatment, as indicated upon ferroptosis induction with RSL3 (**B**) or erastin (**C**). (**D**) Lipid peroxidation was assessed by detection of BODIPY-C11 fluorescence using flow cytometry. Data represent findings from at least three independent experiments. Results are expressed as mean ± SD. Statistical analysis was conducted using one-way ANOVA with Dunnett’s posttest. ** *p* < 0.01, **** *p* < 0.0001.

With the emerging role of the endolysosomal system (ES) in ferroptosis and the potential to manipulate ES function in a variety of ways, we used a panel of ES manipulators and assessed their effect on ferroptosis induction in HCC. We tested several modulators targeting lysosomal cation channels, cathepsins, and autophagy pathways for their potential to modulate sensitivity to ferroptosis induced by erastin or RSL3. Ferrostatin-1 and Z-VAD-FMK were used as positive and negative controls, respectively (Figure 1A). Of note, none of the compounds led to significant cell death induction without ferroptosis inducers (Supplementary Figure S1A) or showed antioxidative properties (Supplementary Figure S1B). Compounds that inhibit autophagy (i.e., Chloroquine, Concanamycin, Siramesine) strongly suppressed ferroptosis induced by erastin and RSL3, confirming the importance of lysosomal degradation pathways in this process, as indicated in the literature [32]. Interestingly, both direct inhibition of TPC2 using sg094 and indirect inhibition via Ned-19 potently inhibited ferroptotic cell death, whereas pharmacological activation of TPC2 with TPC2-A1-N or TPC2-A1-P had no significant effect on ferroptosis sensitivity. Notably, only inhibitors that block TPC2 activation via NAADP (i.e., sg094, Ned-19) were effective in preventing ferroptosis, indicating that NAADP-mediated signaling is critical for TPC2’s role in this pathway. In contrast, inhibition of synthesis of the endogenous TPC2 activator PI(3,5)P_2_ with the PIKfyve inhibitor YM201636 did not alter ferroptosis induction, suggesting that TPC2’s contribution is independent of its PI(3,5)P_2_-mediated regulation. (Figure 1A) In the case of TRPML1, activation of the channel using ML1-SA1 consistently suppressed ferroptosis, while inhibition with EDME led to induction. These findings are consistent with previous reports that TRPML1 is important for ROS sensing and the maintenance of cellular redox homeostasis. (Figure 1A) These observations were confirmed across multiple HCC cell lines, including HepG2, HUH7, and Hep3B, indicating that the effect of manipulation of these ion channels on ferroptosis sensitivity is conserved within the tumor entity (Figure 1B,C).

A major hallmark of ferroptosis is the peroxidation of polyunsaturated fatty acids. Using Bodipy C11, we observed robust lipid peroxide accumulation upon induction of ferroptosis with erastin, RSL3, or glutamate. Lipid ROS levels were potently decreased when ferroptosis inducers were combined with sg094, supporting the notion that TPC2 inhibition exerts protective effects against ferroptotic cell death induction (Figure 1D). Along the line, TRPML1 activation leads to a similar phenotype (Supplementary Figure S1D). Induction of ferroptosis upon treatment with erastin and glutamate was confirmed by combination with the ferroptosis inhibitors ferrostatin-1 or deferoxamine, which abolished cell death (Supplementary Figure S1C).

Taken together, these results suggest a dual regulatory role of lysosomal ion channels: TRPML1 activation protects cells from ferroptosis, whereas NAADP-dependent TPC2 activity appears necessary for ferroptosis induction and sensitivity.

### 3.2. Knockout of TPC2 and TRPML1 Influence Ferroptosis Sensitivity

**Figure 2:**
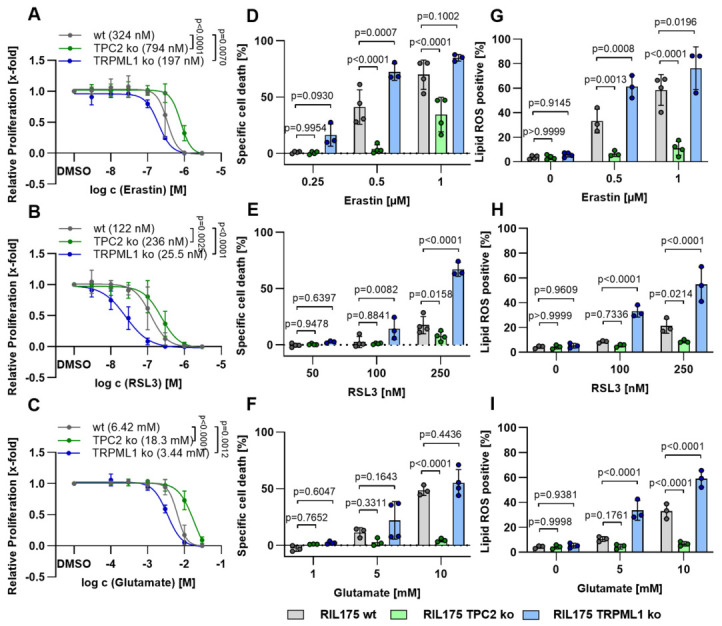
**Knockout of TPC2 and TRPML1 influences ferroptosis sensitivity.** (**A**–**C**) Proliferation of RIL 175 wt, TPC2 KO, and TRPML1 KO cells was assessed by CellTiterBlue^TM^ assay after ferroptosis induction. (**D**–**F**) Specific cell death was determined by propidium iodide staining and flow cytometry after ferroptosis induction. (**G**–**I**) LipidROS levels were assessed by BODIPY™ 581/591 C11 staining and subsequent flow cytometry after ferroptosis induction. Data represent findings from at least three independent experiments. Results are expressed as mean ± SD. Statistical analysis was conducted using one-way ANOVA with Dunnett’s posttest.

To genetically validate these findings based on small molecule inhibitors, we examined ferroptosis sensitivity in knockout cell models by comparing wild-type (wt), TPC2-deficient (TPC2 KO), and TRPML1-deficient (TRPML1 KO) cells. Consistent with our inhibitor studies, TPC2 KO cells exhibited increased resistance to ferroptosis induced by erastin (Figure 2A), RSL3 (Figure 2B), and glutamate (Figure 2C), whereas TRPML1 KO cells were significantly more sensitive to ferroptotic stimuli. Notably, TRPML1 deficiency resulted in a particularly strong sensitization to GPX4 inhibition by RSL3 (Figure 2B), highlighting a critical protective role of TRPML1 in maintaining redox balance under conditions of impaired antioxidant defense. These genetic effects further support our hypothesis and were observed both at the level of overall cell death (Figure 2D–F) and in measurements of lipid peroxidation (Figure 2G–I), suggesting that lysosomal ion channels modulate ferroptosis through modulation of lipid peroxidation.

### 3.3. Fatty Acid Remodeling Renders TPC2 KO Cells Resistant to Ferroptosis

**Figure 3:**
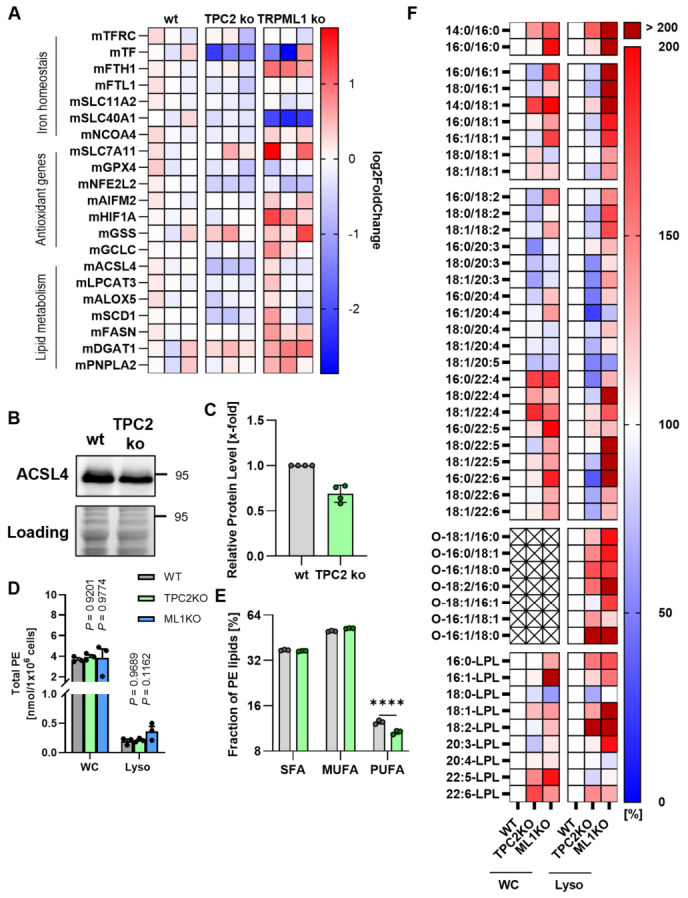
**Fatty acid remodeling renders TPC2 KO cells resistant to ferroptosis.** (**A**) Specific cell death was determined by propidium iodide staining and flow cytometry after ferroptosis induction using erastin or RSL3, respectively. (**B**) Representative image of ACSL4 protein expression assessed by Western blot. Note: LC (**C**) Quantification of B. (**D**) Total amount of PE of whole cell lysates (WC) or isolated lysosomes (Lyso). (**E**) Total amount of PE subfractions (per 10^6^ cells) carrying saturated fatty acids (SFA), monounsaturated fatty acids (MUFA), or polyunsaturated fatty acids (PUFA) in whole cell lysates. The Y-axis scale is log2. (**F**) Total amount of PE subfractions containing either of the indicated fatty acids, normalized to WT whole cells or lysosomes. Ordinary one-way ANOVA + Dunnett’s post hoc test, n = 3, vs. WT control. **** *p* < 0.0001.

To mechanistically assess how TPC2 and TRPML1 influence ferroptosis sensitivity, we focused on three major determinants relevant to the induction of ferroptosis: iron metabolism, cellular oxidative status, and lipid metabolism. Quantitative PCR analysis of known regulatory genes of all three determinants indicates deregulation in lipid metabolism, supposedly via the contribution of ACSL4 modulation in TPC2 KO cells. In TRPML1 KO cells, deregulations in all regulatory pathways are evident, underlining the complexity of its role in cellular homeostasis (Figure 3A). As alterations in lipid metabolism are evident in both channel knock-outs and ACSL4, which has been reported to regulate ferroptosis sensitivity by modulating cellular lipid composition [33], we chose to closely assess lipid metabolism. Investigating ACSL4 expression, we found that the protein level was decreased (Figure 3B,C), which was in line with the qPCR data. As ACSL4-mediated activation of free fatty acids is essential for the incorporation of pro-ferroptotic PUFAs into phospholipids, we analyzed PE levels and composition in whole cells and lysosomes (Figure 3D). There were no significant changes in PE levels of TRPML1 KO cells. Investigating PE composition more closely revealed that the fraction of PUFA-containing PEs is reduced in TPC2 cells. Detailed lipidomic profiling revealed that there are deregulations in channel knock-out cells as compared to wild-type cells, observed in whole cell extracts and lysosomal extracts (Figure 3E,F). We observed a marked decrease in polyunsaturated PE species in lysates from TPC2 knockout cells, whereas TRPML1 knockout cells exhibited changes in the relative proportions of individual PE species without a substantial effect on the overall PUFA fraction within PE. This observation is in line with alterations in ferroptosis sensitivity, especially for TPC2 deletion. A decrease in PUFA-containing PEs provides fewer substrates for lipid peroxidation, driving ferroptosis. Hence, changes in PUFA abundance seem to be relevant for ferroptosis sensitivity, and the observed changes are in line with our initial observation of TPC2 KO increasing ferroptosis resistance.

**Figure 4:**
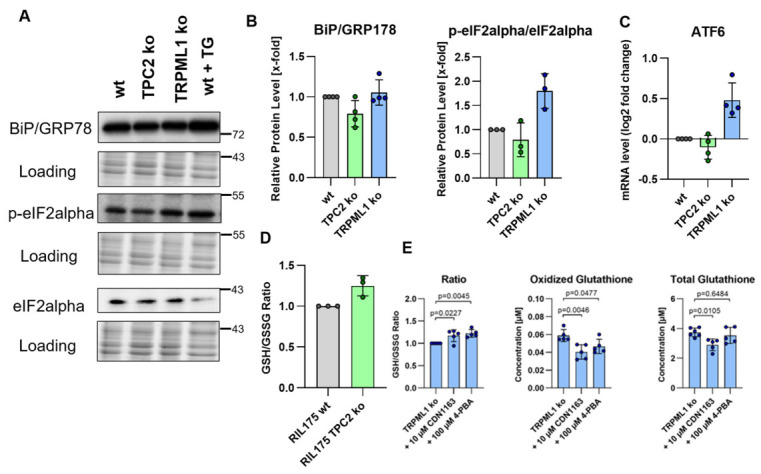
**Ferroptotic signaling pathways and ER stress response.** (**A**) Representative images of protein expression assessed by Western blot. (**B**) Quantification of B. (**C**) The expression of ATF6 was analyzed by qPCR. (**D**,**E**) GSH and GSSG levels were quantified using the GSH/GSSG-Glo assay kit according to the manufacturer’s instructions after treatment as indicated.

Furthermore, we sought to investigate implications of channel knock-outs on cellular redox metabolism, since at least TPRML1 has been published to act as a cellular redox sensor which leads to intracellular calcium release upon ROS prescence [34]. TRPML1 is involved in ER calcium homeostasis, and the ER is a hub for redox processes; dysregulation of ER calcium induces ER stress and protective unfolded protein response. In wt and TPC2 KO cells, none of the ER-stress markers, i.e., BiP/GRP78 and eIF2α, were significantly changed (Figure 4A,B). In TRPML1 KO cells, p-eIF2alpha as well as ATF6 expression was increased (Figure 4A–C). On the other hand, only a slightly increased GSH:GSSG ratio in TPC2 KO cells points towards decreased oxidative stress (Figure 4D). To investigate the impact of ER stress on glutathione metabolism in TRPML1 knockout cells, ER stress was inhibited using either the chemical chaperone 4-PBA or the SERCA activator CDN1163 (Figure 4E). Inhibition of ER stress with both compounds led to an increase in the GSH/GSSG ratio, primarily due to reduced levels of oxidized glutathione (Figure 4E). These findings indicate that ER stress contributes to elevated oxidative stress in TRPML1 knockout cells, which may increase their susceptibility to ferroptosis induction. On the other hand, an increase in expression of eIF and ATF6 points to increased oxidative stress in TRPML1 KO cells, which might contribute to increased ferroptosis sensitivity.

In addition, analysis of genes involved in cellular iron homeostasis revealed a reduction in STEAP3 expression in TPC2 KO cells, whereas STEAP3 expression was increased in TRPML1 KO cells (Supplementary Figure S2A). STEAP3 facilitates the reduction in ferric (Fe^3+^) to ferrous (Fe^2+^) iron within lysosomes, enabling its subsequent transport into the cytosol and thereby contributing to the labile iron pool that promotes ferroptosis. The observed downregulation of STEAP3 in TPC2 KO cells is consistent with reduced ferroptosis sensitivity, while its upregulation in TRPML1 KO cells may support increased iron availability. However, in TRPML1 KO cells, total iron levels remained unchanged, and no significant dysregulation of other key genes involved in iron homeostasis was detected (Supplementary Figure S2B–E), suggesting that compensatory mechanisms may maintain overall iron balance.

Overall, these data suggest that response to ROS generation, but not iron metabolism, mainly contributes to increased ferroptosis sensitivity in TRPML1 KO cells. For TPC2 KO cells, we observed a slight reduction in iron levels, which likely contributes to the ferroptosis-insensitive phenotype. Additionally, lipidomic analysis suggests that a decrease in PUFA-PEs leads to a diminished lipid peroxidation as a driver of ferroptosis resistance in these cells.

### 3.4. Loss of TPC2 Function Alters Cellular Lipid Metabolism

**Figure 5:**
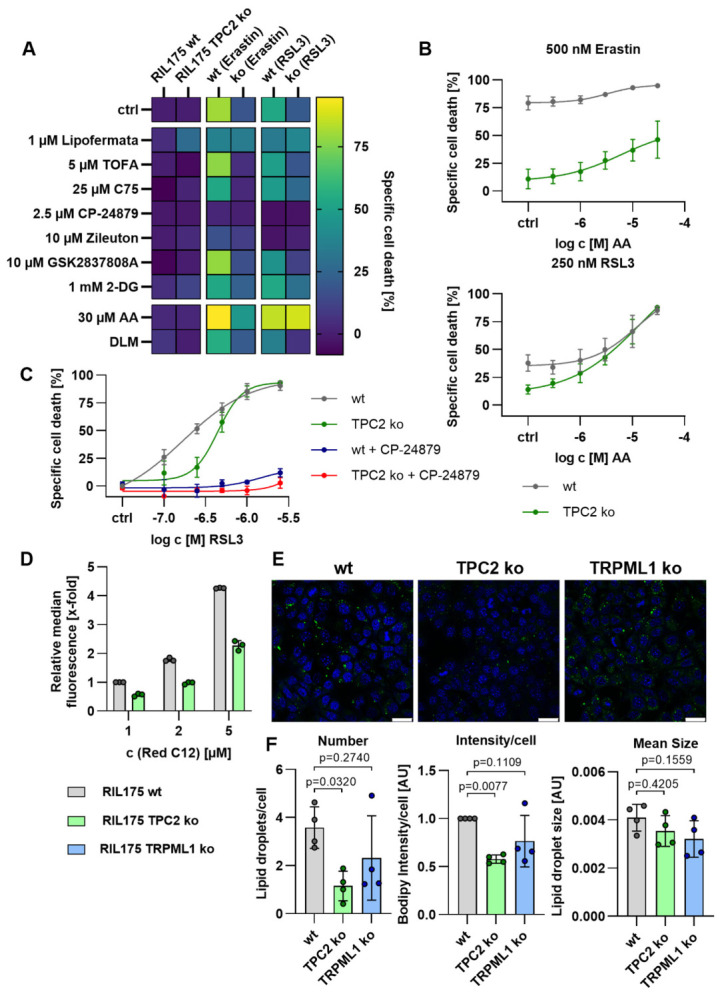
**Loss of TPC2 function alters cellular lipid metabolism.** (**A**–**C**) Specific cell death was determined by propidium iodide staining and flow cytometry after ferroptosis induction using erastin or RSL3 and treatment as indicated, respectively. (**D**) Fatty acid uptake was determined as BODIPY-Red-C12 uptake by flow cytometry. (**E**) Representative confocal images of lipid droplets (green) and nuclei (blue). Scale bar: 10 µm. (**F**) Quantification of (**E**) using ImageJ.

To identify lipid metabolic vulnerabilities that may depend on TPC2 channel function, we performed a metabolism-focused inhibitor screen in RIL-175 wild-type (wt) and TPC2 knockout (TPC2 KO) cells (Figure 5A). Prior to the screen, we confirmed that none of the tested compounds displayed intrinsic antioxidant activity (Supplementary Figure S3A). Because ferroptosis sensitivity is strongly influenced by lipid composition—particularly the balance between saturated/monounsaturated fatty acids (SFA/MUFA) and peroxidizable polyunsaturated fatty acids (PUFAs)—we targeted pathways regulating fatty acid uptake, synthesis, and remodeling. To modulate these pathways, we inhibited fatty acid uptake (Lipofermata), de novo lipogenesis (TOFA, an ACC inhibitor; C75, a fatty acid synthase inhibitor), and PUFA desaturation/remodeling (CP-24879, an inhibitor of FADS1/2). We further targeted lipid peroxidation pathways using Zileuton, a 5-lipoxygenase inhibitor, and modulated cellular metabolic flux with the LDHA inhibitor GSK2837808A and the glycolysis inhibitor 2-deoxy-D-glucose (2-DG). In addition, PUFA availability was directly manipulated by supplementation with arachidonic acid (AA) or by culturing cells in delipidized medium (DLM). Inhibition of de novo lipogenesis with TOFA or C75 did not significantly affect ferroptotic cell death in either wt or TPC2 KO cells. Consistent with this observation, no major differences in fatty acid synthesis pathways were detected between the two genotypes (Supplementary Figure S3B–F). In contrast, increasing the availability of peroxidizable lipids by AA supplementation sensitized cells to ferroptosis. Interfering with lipid peroxidation by inhibition of 5-lipoxygenase with Zileuton markedly reduced ferroptosis (Supplementary Figure S3G), supporting a role for enzymatic lipid peroxidation. Similarly, inhibition of PUFA desaturation and lipid remodeling with the FADS1/2 inhibitor CP-24879 completely blocked RSL3-induced ferroptosis, whereas erastin-induced ferroptosis was only partially suppressed, indicating residual ferroptotic activity despite inhibition of PUFA remodeling (Figure 5C). Importantly, TPC2-dependent differences became evident upon ferroptosis induction. Dose–response analysis revealed that TPC2 KO cells were markedly less sensitive to RSL3-induced cell death than wt cells. Likewise, erastin treatment resulted in significantly higher levels of cell death in wt cells across increasing concentrations of AA, whereas TPC2 KO cells displayed a blunted response (Figure 5C). Consistently, combining RSL3 with increasing AA concentrations further sensitized cells to ferroptosis (Figure 5B). These data indicate that RSL3 sensitivity relies on PUFA balance in phospholipids, while additional factors seem to be relevant upon ferroptosis induction by erastin, in line with the literature [35].

Following analysis of metabolic alterations, we assessed uptake and lipid storage. Uptake of fluorescently labeled fatty acids (Red C12) was decreased in TPC2 KO cells as compared to wt cells, potentially explaining changes in TPC2 KO lipid metabolism and decreased ferroptosis sensitivity (Figure 5D). To characterize lipid storage, we analyzed neutral lipid storage by fluorescence microscopy (Figure 5E), revealing that TPC2 KO cells contained significantly fewer stored lipids per cell compared with wt cells (Figure 5F, left). Moreover, Bodipy 493/503 intensity per cell was decreased in TPC2 KO cells and TRPML1 KO cells (Figure 5F, middle and right). These data suggest that loss of TPC2 alters the intracellular abundance of certain lipid species. Taken together, TPC2 KO leads to a decrease in lipid-Red-C12 uptake, without affecting fatty acid synthesis. We therefore hypothesize that de novo synthesized SFAs/MUFAs contribute more strongly to the cellular fatty acid pool, shifting the balance towards saturation of lipids and subsequently increasing saturation of membrane lipids.

Together, inhibition of highly unsaturated PUFA synthesis with CP24879 or excess supplementation of arachidonic acid, and inhibition of FA uptake, lead to similar ferroptosis sensitivity in TPC2 KO and wt cells. These results indicate that alterations in lipid metabolism are one underlying reason for ferroptosis resistance in TPC2 KO cells.

### 3.5. Kinetic Dependency of Ferroptosis Induction in TPC2 KO Cells

**Figure 6:**
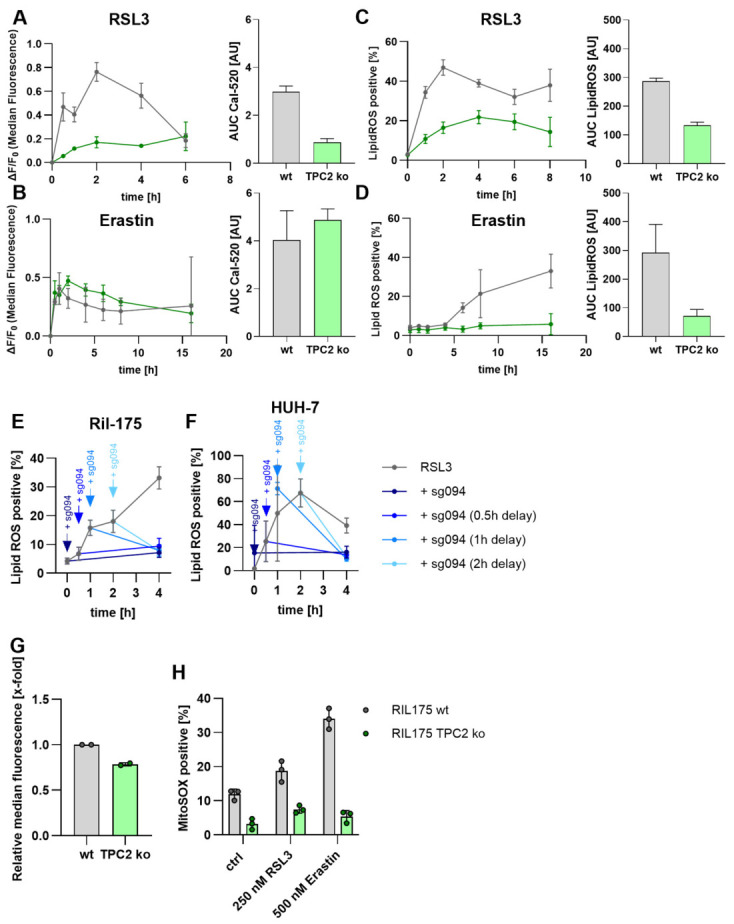
**Kinetic dependency of ferroptosis induction in TPC2 KO cells.** (**A**,**B**) Mean fluorescence intensity of Cal-520 in RIL 175 wt (gray) and TPC2 KO (green) after ferroptosis induction with RSL3 (**A**) or erastin (**B**), n = 2. (**C**,**D**) Lipid reactive oxygen species (ROS) levels in RIL 175 wt and TPC2 KO after ferroptosis induction with RSL3 (**C**) or erastin (**D**). (**E**,**F**) Kinetics of lipid ROS induction over time after addition of sg094 in response to RSL3 measured by flow cytometry. (**G**) Mitochondrial mass assessed by mitotracker staining and flow cytometry. n = 2. (**H**) Determination of mitochondrial superoxides as MitoSOX^TM^ fluorescence by flow cytometry. Data were obtained from a minimum of three independent experiments and are presented as mean ± standard deviation (SD).

To further assess the role of calcium in ROS response, we assessed calcium and lipid ROS levels in a kinetic manner. Based on the role of calcium in ferroptosis and the fact that erastin has been reported to inhibit the mitochondrial calcium regulation, and both ion channels are calcium-permeable channels, we investigate intracellular calcium kinetics upon ferroptosis induction. Both RSL 3 (Figure 6A) and erastin (Figure 6B) lead to an increase in intracellular calcium level within the first 30min. Overall, the increase was greater and longer upon erastin treatment. In TRPML1 KO cells, RSL3 also induced a calcium elevation, whereas erastin only had a minor effect (Supplementary Figure S4A). Of note, lower concentrations of RSL3 or erastin had no effect (Supplementary Figure S4B). In parallel, RSL3 leads to a rapid increase in lipid ROS, especially in wt cells, within the first hour (Figure 6C). Erastin did also increase lipid ROS levels, especially in wt cells, yet with a delay of about 5h (Figure 6D). In TRPML1 KO cells, lipid ROS levels increased more than in the wt cells, regardless of the ferroptosis inducer (Supplementary Figure S4A). To further assess the kinetic relevance of calcium signals, we treated wt Ril-175 (Figure 6E) and HUH-7 (Figure 6F) cells with the TPC2 inhibitor sg094 at several early timepoints after treatment initiation with RSL3 or erastin. Of note, RSL3 does not serve as a direct agonist on TPC2 to induce calcium release (Supplementary Figure S4C). Yet interestingly, inhibition of TPC2 not only prevents lipid ROS formation but can also rescue cell survival after the beginning of lipid ROS elevation. In addition to lipid metabolic alterations promoting ferroptosis surveillance in TPC2 KO cells, this indicates a central role of calcium signals by TPC2 in ferroptosis induction. We hypothesized that lysosomal calcium release by TPC2 upon ferroptosis initiation affects mitochondrial redox state, thereby driving ROS generation and cell death induction. To verify this hypothesis, we assessed mitochondrial mass and stress. Of note, mitochondrial mass is slightly decreased in TPC2 KO cells (Figure 6G). While the positive control antimycin led to a tremendous increase in mitochondrial superoxides (Supplementary Figure S4D), RSL3 led to a moderate increase, and erastin to a substantial increase in wt cells. This increase was completely abolished in TPC2 KO cells (Figure 6H). Of note, a general disruption in cellular calcium signaling using the ionophore calcimycin (Supplementary Figure S5A–C), the SERCA pump inhibitor thapsigagin (Supplementary Figure S5D–H), or inducing ER stress with brefeldin A (Supplementary Figure S5I) or tunicamycin (Supplementary Figure S5J) was not the cause of the differences between wt and ko cells. In sum, we propose that calcium stores seem to be equally loaded, yet upon ferroptosis induction, lysosomal calcium release via TPC2 triggers CICR, resulting in mitochondrial ROS and cell death propagation. This positions TPC2 as a central driver of calcium signaling in the course of ferroptotic cell death.

## 4. Discussion

Cell death evasion is a crucial hallmark of cancer cells that is still a major challenge to overcome. Increasing research suggests that induction of ferroptosis in cancer cells represents a promising therapeutic strategy. As ferroptosis has been closely linked to HCC [19,36,37], with many ferroptosis-regulated genes being involved in the development of hepatocellular carcinoma, and the resistance to first-line drug sorafenib is also inextricably linked to ferroptosis [38] and the lysosomal role in this particular cell death form becomes increasingly evident [38,39,40]. We conducted research on that matter. We show that lysosomal ion channels TRPML1 and TPC2 crucially regulate ferroptosis in HCC in a distinct manner.

The cation channel TPRML1 acts as a protective channel against ferroptosis. Pharmacological activation of TRPML1 suppresses ferroptosis. TRPML1 knockout cells are hypersensitive to ferroptosis, particularly to GPX4 inhibition by RSL3. TRPML1-deficient cells exhibit signs of elevated ER stress and oxidative imbalance. Interestingly, TRPML1 regulates autophagy in response to oxidative stress. Zhang et al. showed that ROS generated by CCCP activates TRPML1, promoting autophagy to clear damaged mitochondria [34]. TRPML1 also affects mitochondrial function directly: its activation increases mitochondrial Ca^2+^ and ROS via lysosome–mitochondria contacts [41], while loss of TRPML1 impairs autophagy, triggering eIF2α-mediated stress responses [28,42]. eIF2α phosphorylation typically occurs as an early event in ER stress, which has also been attributed to loss-of-TRPML1-function in the past [43,44,45]. Consistent with this, Kasitinon et al. show that TRPML1 loss disrupts protein homeostasis in melanoma via mTORC1-driven excess translation, leading to misfolded protein accumulation [46]. In our model, TRPML1 KO may trigger ER calcium depletion and calcium-mediated ER stress, or occur directly through disrupted lysosome–ER calcium crosstalk [26,47,48,49]. Of note, ER stress and oxidative stress are closely linked, as misfolded protein handling generates ROS [50,51,52], which makes it reasonable that ER stress upon TRPML1 KO increases ferroptosis sensitivity. Most likely, deregulation in calcium signaling upon TRPML1 malfunction contributes to the phenotype. We show that calcium levels are strongly deregulated upon TRPML1 KO and greatly affected upon ferroptosis induction. Given that Li et al. [53] showed that TRPML1 lysosomal Ca^2+^ release controls mTORC1 signaling, which is inhibited upon depletion of TRPML1, this links lysosomal Ca^2+^ release with metabolic signaling pathways. In contrast, Xin et al. show that ER luminal Ca^2+^ depletion alters phospholipid composition to reduce lipid peroxidation and confers ferroptosis resistance in cancer cells, linking calcium signaling and lipid peroxidation [54]. In essence, we postulate that TRPML1 KO leads to an e ER-stress response, contributing to the increased ferroptosis sensitivity observed. Furthermore, we suggest that the role of TRPML1 in ferroptosis is a highly interesting field of research that should be investigated in depth in future research.

Lipid peroxidation is an important hallmark in ferroptosis, especially the peroxidation of PUFA-containing phospholipids [55]. We show that loss of TPC2 profoundly alters lipid metabolism, with lipidomic analyses revealing a selective depletion of PUFA-containing phosphatidylethanolamines in TPC2-deficient cells. This finding is an addition to the previously described role of TPC2 in lipid homeostasis, i.e., that in TPC2-deficient mice, endolysosomal trafficking defects were linked to cholesterol accumulation and hyperlipoproteinemia (metabolic dysregulation) and to fatty liver disease (NASH), indicating that TPC2 influences lipid handling in major metabolic organs like the liver [56]. We show that TPC2 KO cells had decreased PUFA-PE levels in favor of MUFA-PE levels, which is known to promote resistance to ferroptosis [35]. Beatty et al. found that conjugated linolenic acids induce lipid peroxidation and ferroptosis in breast cancer cells, which is in accordance with our lipidomics data that show a reduction of 18:2-containing PUFA-PEs [57]. Along the line, Schwab et al. show that inhibition of crucial enzymes SCD and FADS2 adjusts the PUFA:MUFA ratio and affects ferroptosis sensitivity [58]. Additionally, Lee et al. showed that ELOVL5 and FADS1 are required to maintain intracellular levels of arachidonic and adrenic acid and promote ferroptosis [59].

A question that still needs further research to be thoroughly answered is how the TPC2 function is related to changes in lipid metabolism. We found a decreased expression of pro-ferroptotic ACSL4, a master regulator of ferroptosis sensitivity that influences the cellular lipid profile in favor of PUFA-containing phospholipids [33]. This is in line with the literature, showing that genetic or pharmacological inhibition of ACSL4 reduces the abundance of PUFA-containing phospholipids and confers resistance to ferroptosis [3,60]. Interestingly, ACSL4 expression has been linked to calcium signaling. Ren et al. showed that intracellular calcium release leads to ACSL4 upregulation and subsequent changes in lipid metabolism [61]. Our data show that ferroptosis induction leads to an elevation in intracellular calcium levels in wt cells, but not in TPC2 KO cells (Figure 5), which might account for a reduction in ACSL4 expression. Furthermore, Yuan et al., for instance, show that TPC2-mediated lysosomal Ca^2+^ flux regulates lysosomal pH and organelle activity, affecting lipid processing indirectly [62]. Additionally, Xin et al. demonstrated that ER calcium depletion by overexpression of MS4A15 or long-term treatment with thapsigargin renders cancer cells resistant to ferroptosis as a result of depletion of polyunsaturated fatty acids [54]. Remarkably, the difference in intracellular calcium level between wt and TPC2 KO cells is especially evident in RSL3-induced ferroptosis (Figure 5A). There is evidence in the literature showing that RSL3 leads to an intracellular shift in redox balance towards an oxidized cell state. In line with our results, Peng et al. found an increase in intracellular ROS levels, as well as a reduction in GSH levels [63]. This is highly relevant for intracellular NAADP abundance, which is restored in a redox cycle from NAADPH [64]. Hypothesizing that the RSL3-induced shift in redox balance leads to an elevation in NAADP levels would account for the difference in wt and TPC2 KO cells missing in erastin-induced ferroptosis. NAADP, as a direct activator of TPC2, would act as a channel activator and induce calcium release from the lysosomes, further driving cell death, a mechanism that is inhibited in TPC2 KO cells. Additionally, intracellular iron depletion has been reported following TPC2 loss. Fernández et al. showed that TPC2-expressing cells contain higher free iron levels than TPC2-deficient cells. These findings were further validated using Ned-19, an indirect TPC2 inhibitor targeting NAADP. Functionally, TPC2 inhibition reduced ROS generation and subsequent cell death under conditions of exogenous iron overload [65]. Notably, Grimm et al. reported that TPC2 deficiency in mouse embryonic fibroblasts does not impair transferrin uptake [56], implying that other mechanisms are involved. Our data suggest that iron deficiency in TPC2 KO cells may stem from the downregulation of STEAP3 [19]. Given its key role in iron homeostasis, STEAP3 has been investigated in the context of ferroptosis sensitivity. However, its precise role remains ambiguous: Liu et al. reported that STEAP3 knockout confers resistance to ferroptosis [66], whereas Aits et al. found that STEAP3 knockdown increases ferroptosis sensitivity [67]. Our data displays a reduction in STEAP3 mRNA expression upon TPC2 KO and an elevation upon TRPML1 KO, underpinning the distinct roles of both cation channels.

Overall, our findings indicate distinct roles of lysosomal cation channels TPC2 and TRPML1 in ferroptosis of HCC cells. Our experiments indicate that the ER-stress response, rather than iron metabolism, contributes to the heightened ferroptosis sensitivity observed in TRPML1 knockout cells, a finding that would be worthwhile to be thoroughly investigated in future research. In TPC2 knockout cells, a modest reduction in iron levels may play a minor role in their increased resistance; however, lipidomic analyses suggest that the primary factor is a decrease in PUFA-PEs and reduced lipid peroxidation. We suggest that ferroptosis induction leads to a shift in cellular redox balance, resulting in the elevation of NAADP, which in turn activates TPC2. The subsequent cellular calcium increase is a driver for ferroptosis-induced cell death. Hence, TPC2 KO reduces ferroptosis sensitivity.

## 5. Conclusions

Lysosomal ion channels TPC2 and TRPML1 exert distinct roles in regulating ferroptosis sensitivity in hepatocellular carcinoma. Loss of TPC2 function makes HCC cells resistant to ferroptosis by lipid metabolic alterations and modulation of intracellular calcium signaling, whereas TRPML1 knockout increases ferroptosis sensitivity through decreased clearance of ER stress and resulting ROS stress.

## Data Availability

The original contributions presented in this study are included in the article and Supplementary Materials. Further inquiries can be directed to the corresponding authors.

## Supplementary Materials

The following supporting information can be downloaded at: https://www.mdpi.com/article/10.3390/antiox15050618/s1. Supplementary Materials and Methods: Ferric Reducing Antioxidant Power assay, Quantification of GSH/GSSG levels, FerroOrange staining [68]. Figure S1: TPC2 inhibition (or TRPML1 activation) blocks ferroptosis induction, Figure S2: Regulation of iron metabolism response, Figure S3: Regulation of lipid metabolism, Figure S4: Calcium kinetics, Figure S5: Endoplasmatic Reticulum stress response.

## Abbreviations

The following abbreviations are used in this manuscript:

2-DG	2-Deoxy-D-glucose
AA	Arachidonic acid
ACSL	Acyl-CoA synthetase long-chain
ACSL4	Acyl-CoA synthetase long-chain family member 4
ANOVA	Analysis of variance
ATF6	Activating transcription factor 6
BiP/GRP78	Binding immunoglobulin protein/Glucose-regulated protein 78
BODIPY	Boron-dipyrromethene fluorescent dye
BSA	Bovine serum albumin
C75	Fatty acid synthase inhibitor
CCCP	Carbonyl cyanide m-chlorophenyl hydrazone
Cal-520AM	Calcium indicator dye
CT	Threshold cycle
DFO	Deferoxamine
DLM	Delipidized medium
DMEM	Dulbecco’s Modified Eagle Medium
DMSO	Dimethyl sulfoxide
DTT	Dithiothreitol
eIF2α	Eukaryotic initiation factor 2 alpha
ER	Endoplasmic reticulum
ESI	Electrospray ionization
FACS	Fluorescence-activated cell sorting
FCS	Fetal calf serum
FTH1	Ferritin heavy chain 1
GPX4	Glutathione peroxidase 4
GSH	Glutathione
GSSG	Oxidized glutathione
HCC	Hepatocellular carcinoma
HRP	Horseradish peroxidase
HyD	Hybrid detector
ko	Knockout
LDHA	Lactate dehydrogenase A
MRM	Multiple reaction monitoring
MUFA	Monounsaturated fatty acid
NAADP	Nicotinic acid adenine dinucleotide phosphate
NASH	Non-alcoholic steatohepatitis
PBS	Phosphate-buffered saline
PE	Phosphatidylethanolamine
PI(3,5)P_2_	Phosphatidylinositol 3,5-bisphosphate
PIKfyve	Phosphoinositide kinase, FYVE-type zinc finger containing
PMT	Photomultiplier tube
PUFA	Polyunsaturated fatty acid
PVDF	Polyvinylidene difluoride
qPCR	Quantitative polymerase chain reaction
ROS	Reactive oxygen species
RT-qPCR	Reverse transcription quantitative polymerase chain reaction
SD	Standard deviation
SDS-PAGE	Sodium dodecyl sulfate polyacrylamide gel electrophoresis
SFA	Saturated fatty acid
STEAP3	Six-transmembrane epithelial antigen of the prostate 3
TBS-T	Tris-buffered saline with Tween 20
TPC2	Two-pore channel 2
TRPML1	Transient receptor potential mucolipin 1
wt	Wild-type
