# Supplementary material for: Regulation of Ferroptosis Sensitivity in Hepatocellular Carcinoma Cells by Lysosomal Ion Channels TPC2 and TRPML1
Source: Antioxidants (Basel). 2026 May 13;15(5):618. doi: 10.3390/antiox15050618 (PMC13203517; doi:10.3390/antiox15050618)
Supplement: Supplementary file 1 [file antioxidants-15-00618-s001.zip › antioxidants-4259809-supplementary.pdf]

antioxidants

Regulation of Ferroptosis Sensitivity in Hepatocellular Carcinoma Cells by Lysosomal Ion Channels TPC2 and TRPML1

Franz Geisslinger [1], Victoria Gell [1], Finja Witt [2], Dawid Jaślan [3], Christian Grimm [3,4,5], Andreas Koeberle [2,6] and Karin Bartel [1,7,*]

[1] Department of Pharmacy, Ludwig-Maximilians-Universität München, 80539 Munich, Germany
[2] Michael Popp Institute, Center for Molecular Biosciences Innsbruck (CMBI), University of Innsbruck, 6020 Innsbruck, Austria
[3] Walther Straub Institute of Pharmacology and Toxicology, Faculty of Medicine, Ludwig-Maximilians-Universität, 80336 Munich, Germany
[4] Immunology, Infection and Pandemic Research IIP, Fraunhofer Institute for Translational Medicine and Pharmacology ITMP, 80333 Munich, Germany
[5] Department of Pharmacology, Faculty of Medicine, University of Oxford, Oxford OX1 3QT, UK
[6] Institute of Pharmaceutical Sciences and Excellence Field BioHealth, NAWI Graz, University of Graz, Beethovenstraße 8, 8010 Graz, Austria
[7] Cluster for Nucleic Acid Therapeutics Munich (CNAT-M), 80333 Munich, Germany
* Correspondence: karin.bartel@lmu.de

1. Supplementary Materials and Methods

Ferric Reducing Antioxidant Power assay

The Ferric Reducing Antioxidant Power (FRAP) assay allows for the determination of the antioxidant capacity of biological samples in vitro and was performed according to the protocol of Benzie et al. [1] with minor adaptations. Briefly, FRAP reagent was prepared freshly by combining 10 volumes of 300 mM acetate buffer pH 3.5, 1 volume of 10 mM TPTZ (2,4,6-Tri(2-pyridyl)-s-triazine) in 40 mM HCl, and 1 volume of 20 mM iron(III)chloride. The prepared extracts or isolated compounds and varying concentrations of Trolox were incubated with FRAP reagent for 5 min at 37 °C. The reduction of the Fe^{3+}-$(TPTZ)_2$-complex by a possible antioxidative activity is measured colorimetrically with a Tecan SpectraFluor plus microplate reader at 620 nm. A calibration curve with different Trolox concentrations was calculated and antioxidant potential of the extracts and isolated compounds was expressed as absorbance units.

Quantification of GSH/GSSG levels

GSH and GSSG levels were quantified using the GSH/GSSG-Glo assay kit (V6612, Promega) according to the manufacturer's instructions. Cells were seeded in 96 well plates (3×10^3/well) and treated for 24 h. Cells were washed twice in PBS and either lysed in total glutathione lysis reagent (luciferin 1 µl/well, 5X lysis buffer 10 µl/well, H_2O 39 µl/well) or oxidized glutathione lysis reagent (luciferin 1 µl/well, NEM 0.5 µl/well, 5X lysis buffer 10 µl/well, H_2O 38.5 µl/well) and incubated for 5 min while shaking. In parallel, GSH standards were prepared accordingly in concentrations ranging from 0.25 µM to 8 µM. Thereafter, luciferin generation reagent (DTT 1.25 µl/well, GSH-S-transferase 3 µl/well, glutathione reaction buffer 45.75 µl/well) were added to each sample. After 30 min of incubation, 100 µl of luciferin detection reagent were added, plate was equilibrated for 15 min and transferred to white-walled luminescence-compatible 96 well plates. Data were acquired on an Orion II microplate luminometer (Berthold Detection Systems GmbH).

 https://doi.org/10.3390/antiox15050618

Total glutathione and oxidized glutathione were quantified by linear regression from the glutathione standards and GSH/GSSG ratios were calculated as follows:

$$\boldsymbol{GSH : GSSG = \frac{c(total\ GSH) - 2xc(GSSG)}{c(GSSG)}}$$

FerroOrange staining

Ferrous iron levels were quantified with FerroOrange (Dojindo). Cells were seeded in 24 well plates (4×10^4/well) and incubated for 48h. Cells were washed twice with HBSS and stained with 1 μM FerroOrange in HBSS for 30 min at 37°C. Fluorescence intensity was determined on a Tecan plate reader (excitation wavelength: 535 nm, emission wavelength 590 nm). Subsequently, the staining solution was aspirated, cells were washed twice with PBS and stained with crystal violet (0.5% crystal violet in 20% methanol) for 10 min under shaking. Excessive crystal violet was washed off with water and membrane-bound crystal violet was subsequently re-dissolved in trisodium citrate solution (14.7 g/l in 50% ethanol). Absorbance at 550 nm is proportional to the cell number and was employed to normalize FerroOrange fluorescence to the cell number.

1. Benzie, I.F.F. and J.J. Strain, *The Ferric Reducing Ability of Plasma (FRAP) as a Measure of "Antioxidant Power": The FRAP Assay.* Analytical Biochemistry, 1996. **239**(1): p. 70-76.

2. Supplementary Results

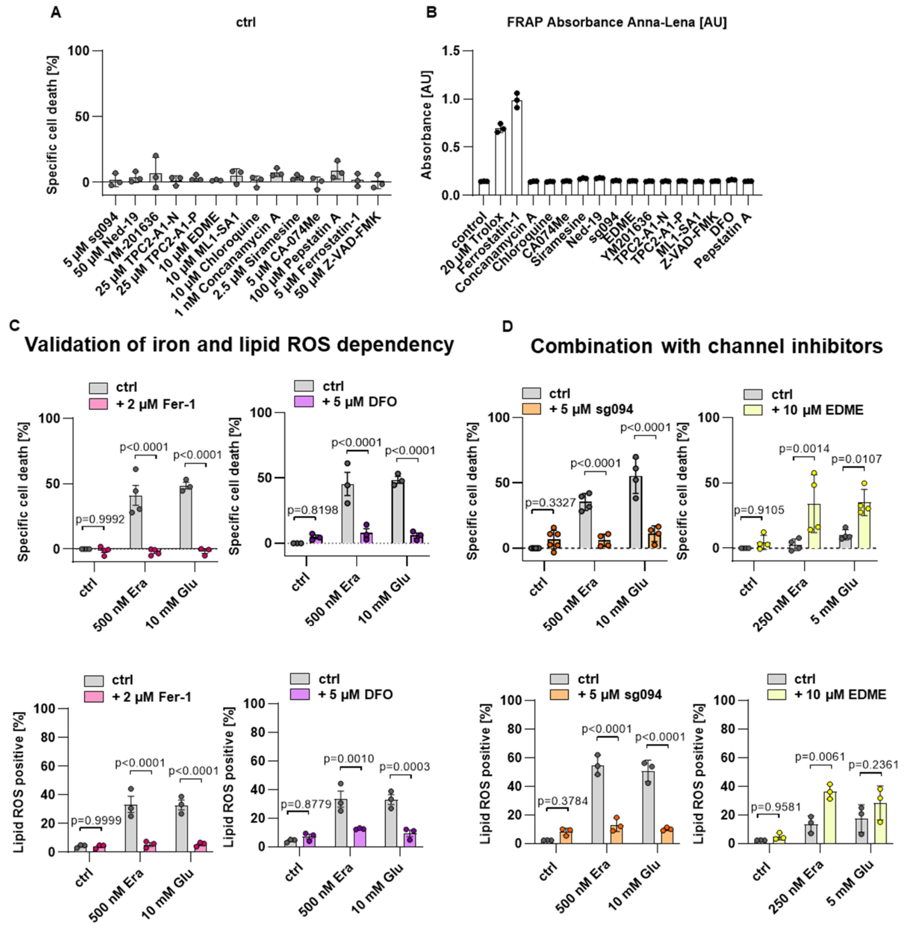

Supplementary Figure S1. TPC2 inhibition (or TRPML1 activation) blocks ferroptosis induction (A) Specific cell death was determined by propidium iodide staining and flow cytometry. **(B)** Antioxidant capacity was determined by FRAP assay. reduction of the Fe^{3+}-(TPTZ)$_2$-complex was measured colorimetrically with a microplate reader at 620 nm. **(C)** Lipid peroxidation was assessed by detection of BODIPY-C11 fluorescence using flow cytometry. **(D)** Specific cell death was determined by propidium iodide staining and flow cytometry Data represent findings from at least three independent experiments. Results are expressed as mean ± SD. Statistical analysis was conducted using one-way ANOVA with Dunnett's posttest.

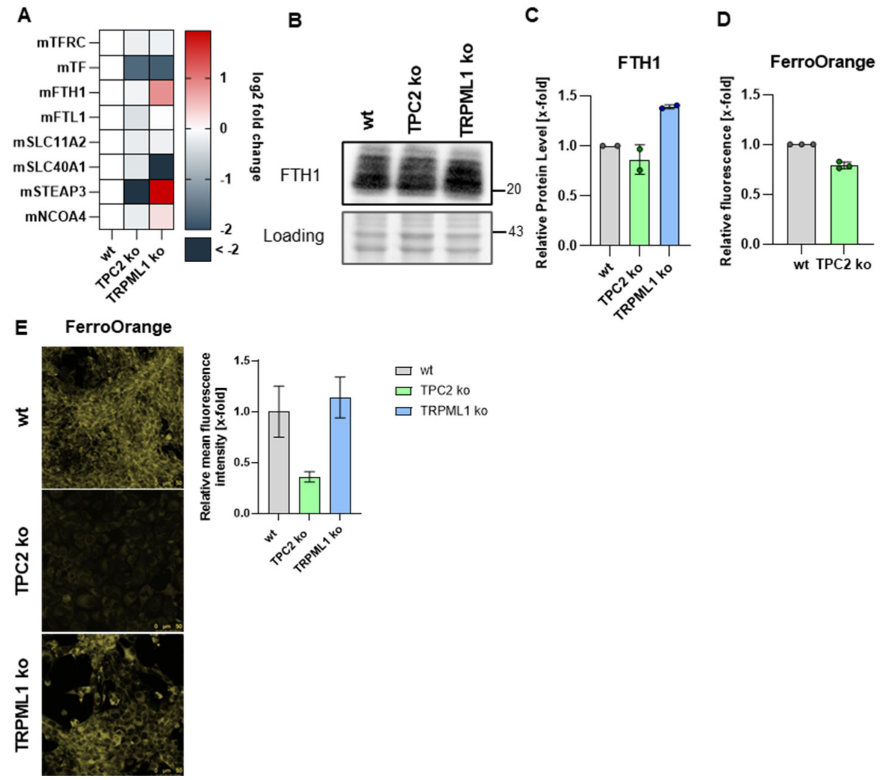

Supplementary Figure S2. Regulation of iron metabolism response (A) The expression of respective genes was analyzed by qPCR. **(B)** Representative images of FTH1 protein expression assessed by Western Blot. **(C)** Quantification of B. **(D)** Liable iron was stained by ferro orange and relative fluorescence intensity was determined by flow cytometry. **(E)** Representative confocal images of ferro orange fluorescence and quantification of images assessed by ImageJ.

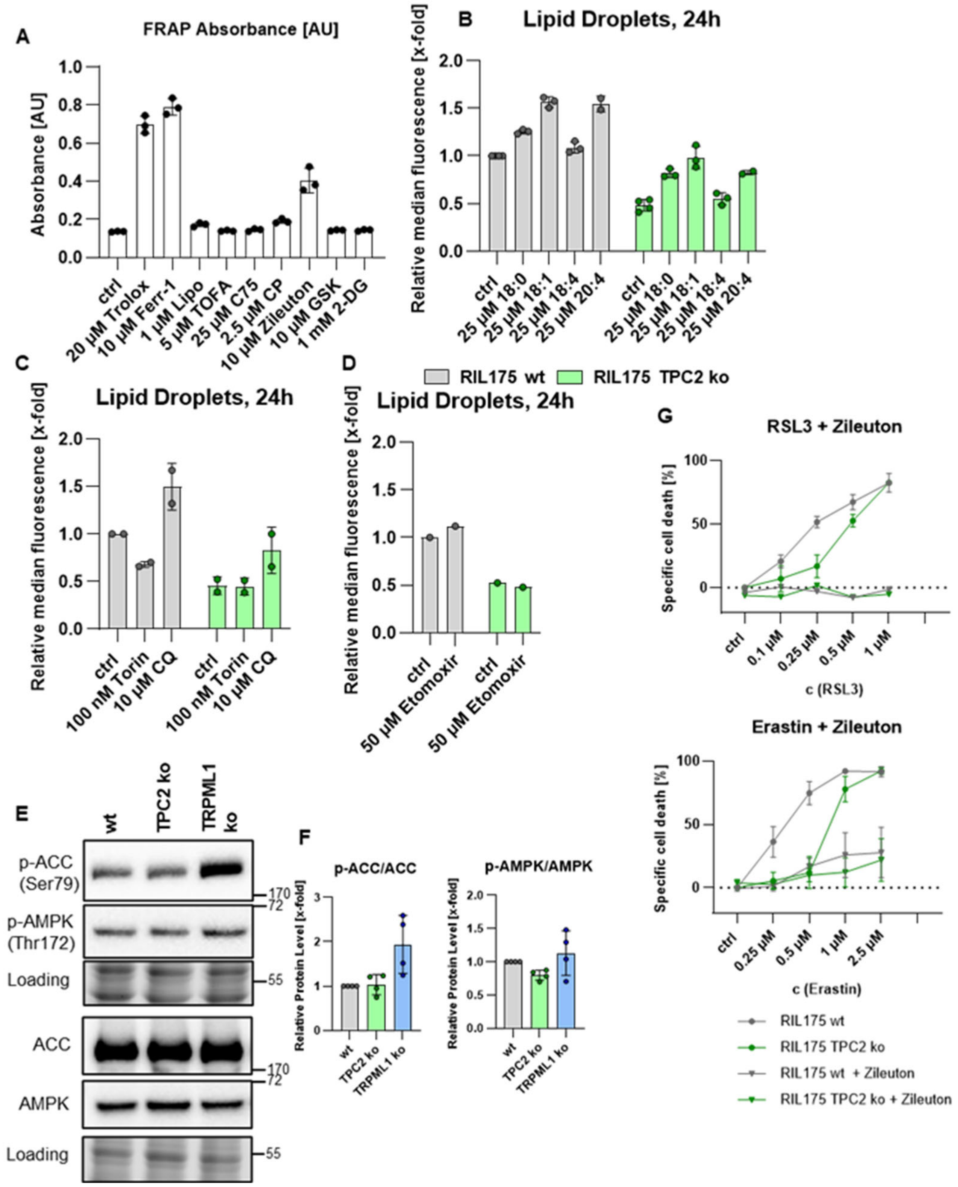

Supplementary Figure S3. Regulation of lipid metabolism (A) Antioxidant capacity was determined by FRAP assay. reduction of the Fe^{3+}-$(TPTZ)_2$-complex was measured colorimetrically with a microplate reader at 620 nm. **(B–D)** Relative mean fluorescence intensity of lipid droplets was assessed by Bodipy 493/503 staining and subsequent flow cytometry. **(E)** Representative images of protein expression assessed by Western Blot. **(F)** Quantification of E indicating the ratio of phosphorylated to total protein. **(G)** Specific cell death was determined by propidium iodide staining and flow cytometry.

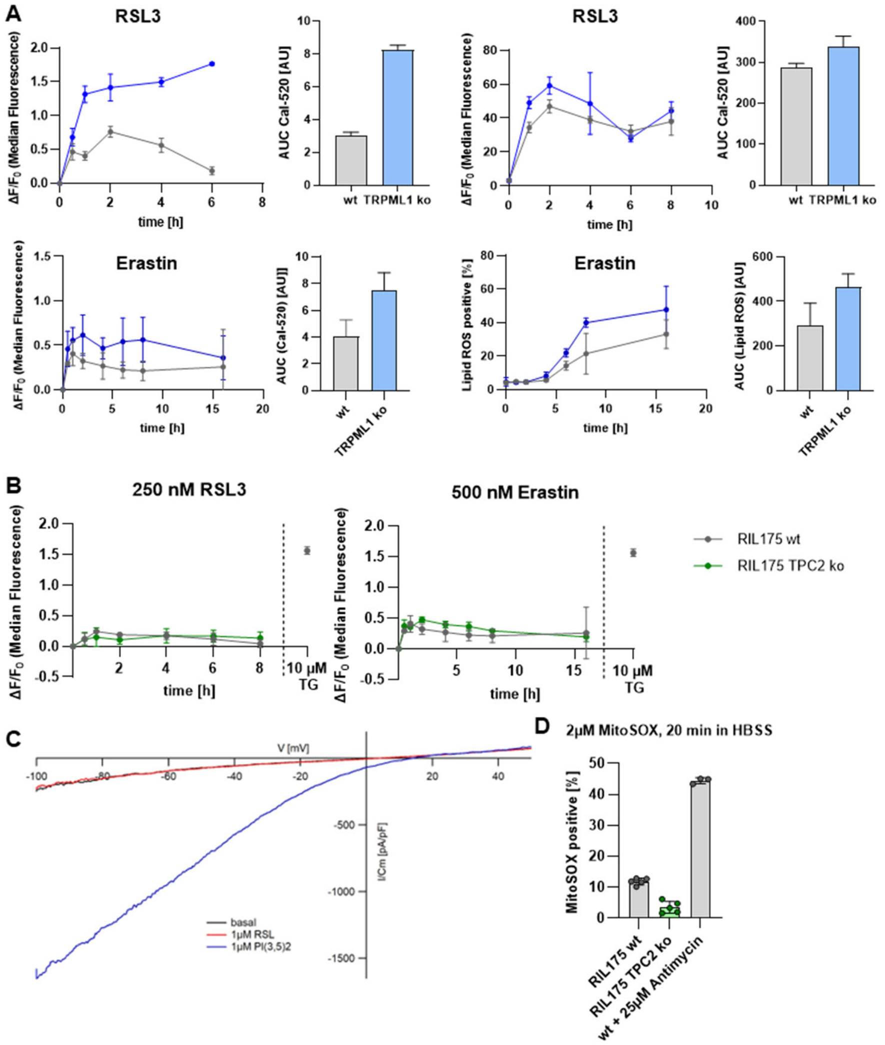

Supplementary Figure S4. Calcium kinetics (A) Mean fluorescence measurements in RIL 175 wt and TRPML1 ko. Lipid reactive oxygen species (ROS) levels in RIL 175 wt and TRPML1 ko. **(B)** Intracellular calcium levels determined as Cal-520 fluorescence assessed by flow cytometry. **(C)** Exemplary current of whole endosome patch clamp after TPC2 activation using PI(3,5)P_2 or RSL3. **(D)** Determination of mitochondrial superoxides as MitoSOX™ fluorescence signal detected by flow cytometry.

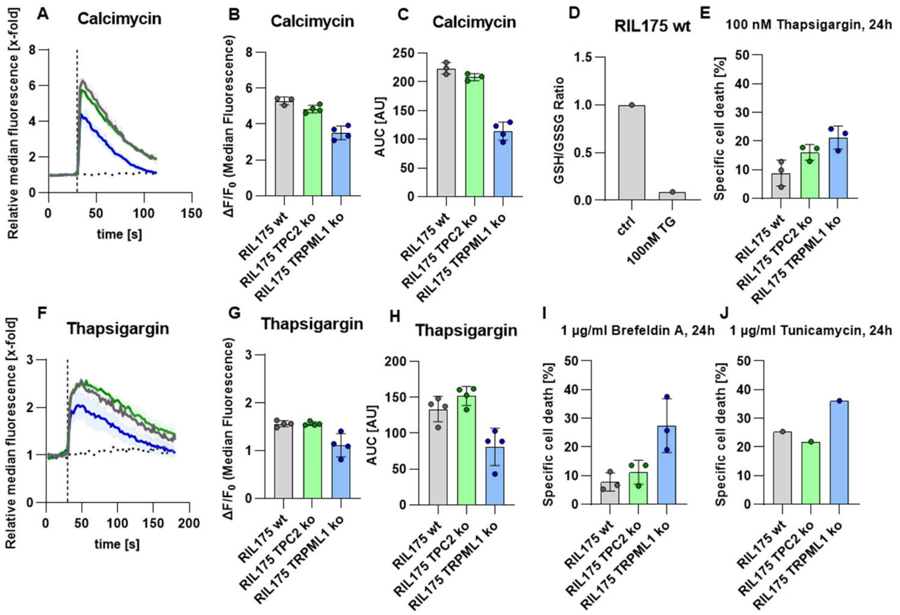

Supplementary Figure S5. Endoplasmatic Reticulum stress response (A-C) Intracellular calcium levels determined as Cal-520 fluorescence assessed by flow cytometry. **(D)** GSH and GSSG levels were quantified using the GSH/GSSG-Glo assay kit according to the manufacturer's instructions. **(E-H)** Intracellular calcium levels determined as Cal-520 fluorescence assessed by flow cytometry. **(I-J)** Specific cell death was determined by propidium iodide staining and flow cytometry.

https://doi.org/10.3390/antiox15050618
